# Dataset on posttraumatic growth in women survived breast cancer

**DOI:** 10.1016/j.dib.2020.106468

**Published:** 2020-10-27

**Authors:** Anna G. Faustova

**Affiliations:** Ryazan State Medical University, Russia

**Keywords:** Breast cancer, remission, posttraumatic stress, posttraumatic growth, purpose in life, Russia

## Abstract

Posttraumatic growth is a set of positive psychological changes that happened to a person after he/she has been exposed to psychological trauma. Cancer diagnosis and treatment could cause severe psychological trauma. Women diagnosed with breast cancer have to deal with not only physical outcomes but also with psychosocial ones. After a complete remission is confirmed, some of them develop new meaning and purpose in life, change a job, improve relationships, etc. In this study, we assessed the characteristics of posttraumatic growth in 30 women (mean age – 55 years) with breast cancer in complete remission. We used the Posttraumatic Growth Inventory, the Purpose-In-Life Test, and the Impact of Event Scale-Revised. In this article, the raw data, summed subscale scores, descriptive statistics, and results of the correlational analysis are presented. The dataset may be used for making cross-cultural comparisons and for a further in-depth examination of positive experience in cancer survivors.

**Specifications Table**

**Table:** 

Subject	Psychology
Specific subject area	Clinical and Health Psychology, Psychotraumatology
Type of data	Tables
How data were acquired	Self-report based scales and inventories were administered to the participants.
Data format	Raw Summed subscale scores Analyzed
Parameters for data collection	Women diagnosed with breast cancer took part in this research project six months after complete remission is confirmed. All the participants signed the Informed Consent Form.
Description of data collection	The researcher examined the participants individually. Before administering the standardized tests, the researcher asked women a few questions about their experience upon the diagnosis. After the conversation, women filled out the Posttraumatic Growth Inventory, the Purpose-In-Life Test, and the Impact of Event Scale-Revised. Data were collected hardcopy.
Data source location	Institutions: Ryazan State Medical University, Ryazan Region Clinical Oncology Center. City: Ryazan. Country: Russian Federation.
Data accessibility	Raw (per-item) data are uploaded to Mendeley Data. Direct URLs to data:http://dx.doi.org/10.17632/5f4rfjjh3d.1http://dx.doi.org/10.17632/vry9nmtg8g.1http://dx.doi.org/10.17632/bs7283bg9m.1 Summed subscale scores and analyzed data are available with the article.

**Value of the Data**

• Being diagnosed with any type of cancer and undergoing cancer treatment are very stressful events. However, surviving cancer may have some positive psychological outcomes called “posttraumatic growth” [1], [2], [3]. Body image disturbances, losing the sense of femininity, anxiety, depression, and poor quality of life are the most studied psychological aspects of having breast cancer in women [4], [5], [6].
• Focusing on positive psychological outcomes of fighting cancer may benefit both doctors and patients to establish more trustful relationships. Further use of the data to reveal psychological mechanisms of successful coping may help to promote optimism and hope in women who are newly diagnosed with breast cancer.
• The data are of considerable practical importance since it may be used for making some cross-cultural comparisons. Despite the relatively small sample size, the data generally reflect the main characteristics of female citizens of Central Russia.
• Cancer researchers may consider the dataset as an initial pack for a further in-depth examination of what people experience upon the cancer diagnosis and treatment. They also may use the data for making comparisons with samples consisted of patients with other types of cancer.
• Clinical psychologists and specialists in cancer rehabilitation may find this dataset useful for developing evidence-based programs of psychosocial support for those who are finishing cancer treatment.

## 1 Data Description

Table 1 contains social, demographic, and medical data about the participants. For each participant, her code, age at the time of diagnosis, age at the time of examination, educational level, employment status, and marital status are shown. The column “Details of diagnosis” includes the precise localization of a tumor (left/right breast) as well as the stage of malignancy in accordance with the TNM classification. The data for each participant are timestamped.

**Table 1:** Social, demographic, and medical data.

Participant's code	Age at the time of diagnosis	Age at the time of examination	Time stamp	Educational level	Employment status	Marital status	Details of diagnosis
1	57	60	7 Oct 2019	Post-secondary	Employed	Widowed	T2N1M0, right breast
2	60	62	7 Oct 2019	Bachelor	Retired	Married	T2N0M0, right breast
3	46	49	11 Oct 2019	Master	Employed	Married	T2N1M0, left breast
4	49	51	11 Oct 2019	Master	Employed	Serious relationships	T2N1M0, left breast
5	45	47	11 Oct 2019	Bachelor	Employed	Divorced	T2N0M0, left breast
6	49	51	14 Oct 2019	Bachelor	Employed	Married	T1N0M0, left breast
7	45	47	18 Oct 2019	Master	Self-employed	Married	T1N1M0, right breast
8	49	52	18 Oct 2019	Master	Self-employed	Married	T2N0M0, left breast
9	50	52	21 Oct 2019	Master	Currently unemployed	Widowed	T2N0M0, right breast
10	54	57	21 Oct 2019	Post-secondary	Employed	Divorced	T2N0M0, left breast
11	53	56	25 Oct 2019	Master	Employed	Married	T1N0M0, right breast
12	65	68	25 Oct 2019	Master	Retired	Married	T2N0M0, left breast
13	64	66	25 Oct 2019	Bachelor	Retired	Married	T2N0M0, left breast
14	37	41	28 Oct 2019	Master	Employed	Serious relationships	T2N1M0, left breast
15	51	54	28 Oct 2019	Master	Employed	Married	T2N0M0, left breast
16	43	45	1 Nov 2019	Master	Employed	Married	T1N0M0, right breast
17	36	39	1 Nov 2019	Master	Employed	Married	T2N0M0, right breast
18	55	57	1 Nov 2019	Bachelor	Currently unemployed	Divorced	T2N0M0, left breast
19	52	54	8 Nov 2019	Master	Self-employed	Serious relationships	T1N0M0, left breast
20	58	61	8 Nov 2019	Bachelor	Retired	Married	T2N1M0, left breast
21	58	60	11 Nov 2019	Master	Employed	Widowed	T2N0M0, right breast
22	59	62	11 Nov 2019	Post-secondary	Retired	Married	T2N1M0, right breast
23	60	62	11 Nov 2019	Master	Employed	Married	T2N0M0, left breast
24	55	59	15 Nov 2019	Bachelor	Employed	Divorced	T2N1M0, left breast
25	67	69	15 Nov 2019	Bachelor	Retired	Widowed	T2N0M0, right breast
26	70	73	18 Nov 2019	Post-secondary	Retired	Widowed	T2N1M0, left breast
27	59	61	22 Nov 2019	Master	Employed	Married	T2N0M0, left breast
28	49	53	22 Nov 2019	Master	Employed	Married	T2N0M0, right breast
29	47	50	25 Nov 2019	Master	Employed	Married	T1N0M0, left breast
30	55	57	25 Nov 2019	Bachelor	Currently unemployed	Widowed	T2N1M0, left breast

Table 2 contains the summed subscale scores collected by administering the Posttraumatic Growth Inventory (a copy of the questionnaire in English is provided as a supplementary file). For each participant, her code and individual results are represented. The raw data (the actual item scores) were uploaded to Mendeley Data [7].

**Table 2:** Summed subscale scores obtained using the posttraumatic growth inventory.

Participant's code	Subcales of the Posttraumatic Growth Inventory				
	Relating to others	New Possibilities	Personal Strength	Spiritual Change	Appreciation of life
1	12	15	12	4	8
2	16	4	14	7	7
3	22	11	9	9	10
4	21	17	13	4	6
5	8	15	8	6	8
6	19	15	16	6	7
7	28	18	7	3	6
8	17	9	12	4	6
9	23	16	10	6	7
10	9	11	7	7	13
11	28	19	16	6	14
12	15	11	8	4	7
13	21	6	9	3	6
14	23	21	19	9	15
15	26	15	12	8	10
16	15	10	5	4	9
17	17	13	9	8	12
18	31	20	17	10	15
19	2	1	0	0	0
20	34	23	19	10	14
21	16	0	0	0	2
22	15	8	12	9	13
23	13	8	7	6	12
24	15	10	13	9	14
25	11	0	7	1	6
26	14	3	4	0	5
27	24	14	15	9	12
28	25	19	18	7	14
29	29	19	19	8	15
30	20	14	12	2	12

Table 3 includes the summed scores collected by administering both the Purpose-In-Life Test and the Impact of Event Scale (copies of these questionnaires in English are provided as supplementary files). For each participant, her code and obtained results are provided. The raw data (the actual item scores) were uploaded to Mendeley Data [8,9].

**Table 3:** Summed subscale/scale scores obtained using the purpose-in-life test and the impact of event scale-revised.

Participant's code	Purpose-in-life test	Subscales of the impact of event scale-revised		
		Intrusion	Avoidance	Hyperarousal
1	72	8	22	17
2	99	17	19	11
3	93	19	16	21
4	103	19	24	13
5	80	17	18	17
6	81	11	16	11
7	109	18	20	23
8	102	15	18	15
9	86	18	17	17
10	87	14	22	13
11	110	5	1	3
12	70	32	32	23
13	112	9	12	4
14	126	13	9	4
15	98	16	16	11
16	113	9	9	0
17	117	3	14	1
18	123	15	26	1
19	140	3	0	1
20	122	4	3	2
21	114	6	21	4
22	107	19	23	14
23	103	27	17	17
24	106	25	20	23
25	96	10	6	6
26	76	4	5	0
27	80	32	21	24
28	115	8	7	1
29	129	6	1	3
30	112	10	9	5

Table 4 includes the analyzed data obtained after calculating both measures of central tendency (Mean) and measures of variability (Dispersion, Standard Deviation). Many variables in this dataset are either binomial or count, which means they are not distributed normally in the population. Moreover, recent research suggests that posttraumatic growth is typically negatively skewed [10]. Thus, if data are utilized in multivariate analysis, users should pay close attention to the assumptions, such as the normal distribution of the data.

**Table 4:** Descriptive statistics of the data.

Test/Inventory	Scale	Mean	Dispersion	Standard Deviation
Age	Age at the time of diagnosis	53.233	66.667	8.165
	Age at the time of examination	55.833	64.971	8.060
Purpose-In-Life Test	Purpose-in-Life	102.700	318.010	17.832
Impact of Event Scale	Intrusion	13.733	64.891	8.055
	Avoidance	14.800	66.993	8.184
	Hyperarousal	10.166	66.674	8.128
Posttraumatic Growth Inventory	Relating to Others	18.966	59.964	7.346
	New Possibilities	12.166	40.557	6.368
	Personal Strength	10.966	27.136	5.209
	Spiritual Change	5.633	9.481	3.079
	Appreciation of Life	9.500	16.534	4.066

Table 5 contains the analyzed data obtained by calculating Pearson correlations. There are significant negative correlations between the measure of purpose in life and intrusion (-0.458, p<0.05), avoidance (-0.502, p<0.01), and hyperarousal (-0.604, p<0.01). One possible limitation here is that these associations may be overestimated due to the small size of the sample. There are no significant correlations between the parameters of posttraumatic growth and the subscales of the Impact of Event Scale-Revised.

**Table 5:** Results of the correlational analysis.

	Purpose-in-Life	Intrusion	Avoidance	Hyperarousal
Purpose-in-Life	1.000	−0.458*	−**0.502**⁎⁎	**−0.604**⁎⁎
Relating to others	0.277	−0.031	−0.117	−0.124
New Possibilities	0.118	0.033	−0.017	0.031
Personal Strength	0.140	0.061	−0.085	−0.042
Spiritual Change	0.090	0.312	0.174	0.192
Appreciation of Life	0.210	0.117	−0.073	−0.058

⁎ Significant correlations (p<0.05)

⁎⁎ Significant correlations (p<0.01)

## 2 Experimental Design, Materials, and Methods

*Participants.* The sample consisted of 30 Caucasian women (aged from 39 to 73, with a mean age of 55 years) with breast cancer who achieved a complete remission. They did not have any other types of cancer as comorbid. All women underwent a mastectomy and from 10 to 40 courses of chemotherapy. They did not take any psychotropic medications (not during cancer treatment, nor after the treatment is completed). They are comparable in socio-demographic characteristics such as marital and family status, educational level, and employment status. Their socioeconomic status may be characterized as middle-income. The social, demographic, and medical data are provided in detail in Table 1.

*Experimental Design.* The sample is described as a single cohort based on the time of remission onset. All the participants were approached one by one when they came for a routine check to the outpatient department of the Ryazan Region Clinical Oncology Center. Each participant signed the Informed Consent Form. Before administering the standardized tests, all women answered a few open-ended questions about their experience upon the diagnosis. The purpose of these questions was to get participants involved in the research project and to develop the motivation to participate further. After a brief conversation, the participants filled out three standardized measures. Data were collected hardcopy.

*Standardised measures.*

1. Posttraumatic Growth Inventory. This inventory was developed by R.G. Tedeschi and L.G. Calhoun (1996) [1]. The Russian adaptation was made by M.Sh. Magomed-Eminov (2004). It consists of 21 items and includes the following subscales: Relating to others, New Possibilities, Personal Strength, Spiritual Change, and Appreciation of Life. Each statement should be rated on a 6-point Likert scale where (0) – no changes happened; (1) – a very small degree of changes; (2) – a small degree; (3) – a moderate degree; (4) – a great degree; (5) – a very great degree of changes.

2. Impact of Event Scale-Revised. This revised version of the Impact of Event Scale (by M. Horowitz, N. Wilner, and W. Alvarez, 1979) was created by D.S. Weiss and C.R. Marmar (1996) [11]. The Russian adaptation was made by N.V. Tarabrina (2001). It includes 22 items summarized into three subscales, such as Intrusion, Avoidance, and Hyperarousal. All statements are rated using a 5-point scale where (0) – not at all; (1) – a little bit; (2) – moderately; (3) – quite a bit; (4) – extremely.

3. Purpose-In-Life Test. This test was developed by J.C. Crumbaugh and L.T. Maholick (1976) [12]. The Russian adaptation was made by D.A. Leontiev (1988). It consists of 20 statements that should be rated using a 7-point Likert scale.

Microsoft Excel was used to calculate descriptive statistics and Pearson correlations.

## Ethics Statement

The School of Clinical Psychology at the Ryazan State Medical University (Ryazan, Russia) provided us with the ethical approval for this research project. All the participants gave informed consent for participating in the research project.

## Declaration of Competing Interest

The author declare that she has no known competing financial interests or personal relationships which have, or could be perceived to have, influenced the work reported in this article.

## Appendix Supplementary materials

Image, application 1

Image, application 2

Image, application 3
