# Supplementary material for: Dataset on posttraumatic growth in women survived breast cancer
Source: Data Brief. 2020 Oct 27;33:106468. doi: 10.1016/j.dib.2020.106468 (PMC7644875; doi:10.1016/j.dib.2020.106468)
Supplement: Supplementary file 1 [file mmc1.docx]

*Supplementary file #1*

**POSTTRAUMATIC GROWTH INVENTORY**

**R.G. Tedeschi and L.G. Calhoun**

For each of the statements below indicate the degree to which this change occurred in your life as a result of your crisis, using the following scale.

(0) – no changes happened;

(1) – a very small degree of changes;

(2) – a small degree;

(3) – a moderate degree;

(4) – a great degree;

(5) – a very great degree of changes.

1. My priorities what is important in life.
2. An appreciation for the value of my own life.
3. I developed new interests.
4. A feeing of self-reliance.
5. A better understanding of spiritual matters.
6. Knowing that I can count on people in times of trouble.
7. I established a new path for my life.
8. A sense of closeness with others.
9. A willingness to express my emotions.
10. Knowing I can handle difficulties.
11. I am able to do better things with my life.
12. Being able to accept the way things work out.
13. Appreciating each day.
14. New opportunities are available which would not have been otherwise.
15. Having compassion for others.
16. Putting effort into my relationships.
17. I am more likely to try to change things, which need changing.
18. I have a stronger religious faith.
19. I discovered that I am stronger than I thought I was.
20. I learned a great deal about how wonderful people are.
21. I accept needing others.

**Reference:**

R.G. Tedeschi, L.G. Calhoun, The Posttraumatic Growth Inventory: Measuring the Positive Legacy of Trauma, J. Trauma. Stress. 9(3) (1996) 455-471. doi: 10.1007/BF02103658.
