# Supplementary material for: Dataset on posttraumatic growth in women survived breast cancer
Source: Data Brief. 2020 Oct 27;33:106468. doi: 10.1016/j.dib.2020.106468 (PMC7644875; doi:10.1016/j.dib.2020.106468)
Supplement: Supplementary file 2 [file mmc2.docx]

*Supplementary file #2*

**IMPACT OF EVENTS SCALE-Revised**

**D.S. Weiss and C.R. Marmar**

Below is a list of difficulties people sometimes have after stressful life events. Please read each item, and then indicate how distressing each difficulty has been for you DURING THE PAST SEVEN DAYS. How much have you been distressed or bothered by these difficulties?

(0) – not at all; (1) – a little bit; (2) – moderately; (3) – quite a bit; (4) – extremely.

1. Any reminder brought back feelings about it
2. I had trouble staying asleep.
3. Other things kept making me think about it.
4. I felt irritable and angry.
5. I avoided letting myself get upset when I thought about it or was reminded of it.
6. I thought about it when I did not mean to.
7. I felt as if it had not happened or was not real.
8. I stayed away from reminders of it.
9. Pictures about it popped into my mind.
10. I was jumpy and easily startled.
11. I tried not to think about it.
12. I was aware that I still had a lot of feelings about it, but I did not deal with them.
13. My feelings about it were kind of numb.
14. I found myself acting or feeling like I was back at that time.
15. I had trouble falling asleep.
16. I had waves of strong feelings about it.
17. I tried to remove it from my memory.
18. I had trouble concentrating.
19. Reminders of it caused me to have physical reactions, such as sweating, trouble breathing, nausea, or a pounding heart.
20. I had dreams about it.
21. I felt watchful and on-guard.
22. I tried not to talk about it.

**Reference:**

D.S. Weiss, The Impact of Event Scale-Revised, in: J.P. Wilson, T.M. Keane (Eds.), Assessing psychological trauma and PTSD: a practitioner's handbook, Guilford Press, New York, 2007, pp. 168-189.
