# Supplementary material for: Dataset on posttraumatic growth in women survived breast cancer
Source: Data Brief. 2020 Oct 27;33:106468. doi: 10.1016/j.dib.2020.106468 (PMC7644875; doi:10.1016/j.dib.2020.106468)
Supplement: Supplementary file 3 [file mmc3.docx]

*Supplementary file #3*

**PURPOSE IN LIFE TEST**

**J.C. Crumbaugh and L.T. Maholick**

Read each of the statements below and mark the number (1 to 7) next to each statement that is most true for you right now.

1. I am usually:

bored 1 --- 2 --- 3 --- 4 --- 5 --- 6 --- 7 enthusiastic.

2. Life to me seems:

completely routine 1 --- 2 --- 3 --- 4 --- 5 --- 6 --- 7 always exciting.

3. In life I have:

no goals or aims 1 --- 2 --- 3 --- 4 --- 5 --- 6 --- 7 clear goals and aims.

4. My personal existence is:

utterly meaningless, without purpose 1 --- 2 --- 3 --- 4 --- 5 --- 6 --- 7 purposeful and meaningful.

5. Every day is:

exactly the same 1 --- 2 --- 3 --- 4 --- 5 --- 6 --- 7 constantly new and different.

6. If I could choose, I would:

prefer never to have been born 1 --- 2 --- 3 --- 4 --- 5 --- 6 --- 7 want 9 more lives just like this one.

7. After retiring, I would:

loaf completely the rest of my life 1 --- 2 --- 3 --- 4 --- 5 --- 6 --- 7 do some of the exciting things I've always wanted to.

8. In achieving life goals I’ve:

made no progress whatever 1 --- 2 --- 3 --- 4 --- 5 --- 6 --- 7 progressed to complete fulfillment.

9. My life is:

empty, filled only with despair 1 --- 2 --- 3 --- 4 --- 5 --- 6 --- 7 running over with exciting things.

10. If I should die today, I’d feel that my life has been:

completely worthless 1 --- 2 --- 3 --- 4 --- 5 --- 6 --- 7 very worthwhile.

11. In thinking of my life, I:

often wonder why I exist 1 --- 2 --- 3 --- 4 --- 5 --- 6 --- 7 always see reasons for being here.

12. As I view the world in relation to my life, the world:

completely confuses me 1 --- 2 --- 3 --- 4 --- 5 --- 6 --- 7 fits meaningfully with my life.

13. I am a:

very irresponsible person 1 --- 2 --- 3 --- 4 --- 5 --- 6 --- 7 very responsible person.

14. Concerning freedom to choose, I believe humans are:

completely bound by limitations of heredity and environment 1 --- 2 --- 3 --- 4 --- 5 --- 6 --- 7 totally free to make all life choices.

15. With regard to death, I am:

unprepared and frightened 1 --- 2 --- 3 --- 4 --- 5 --- 6 --- 7 prepared and unafraid.

16. Regarding suicide, I have:

thought of it seriously as a way out 1 --- 2 --- 3 --- 4 --- 5 --- 6 --- 7 never given it a second thought.

17. I regard my ability to find a purpose or mission in life as:

practically none 1 --- 2 --- 3 --- 4 --- 5 --- 6 --- 7 very great.

18. My life is:

out of my hands and controlled by external factors 1 --- 2 --- 3 --- 4 --- 5 --- 6 --- 7 in my hands and I’m in control of it.

19. Facing my daily tasks is:

a painful and boring experience 1 --- 2 --- 3 --- 4 --- 5 --- 6 --- 7 a source of pleasure & satisfaction.

20. I have discovered:

no mission or purpose in life 1 --- 2 --- 3 --- 4 --- 5 --- 6 --- 7 a satisfying life purpose.

**References:**

[1] G. Davies, D. Klaassen, A. Längle, Purpose in Life Test, in: A.C. Michalos (eds), Encyclopedia of Quality of Life and Well-Being Research, Springer, Dordrecht, 2014, pp.5238-5243. <https://doi.org/10.1007/978-94-007-0753-5_2336>

[2] Meaning and purpose in Russian, Swiss, and American adolescents. http://www.leeds.ac.uk/educol/documents/000000483.htm, 2020 (accessed 10 October 2020).
